# Transplant characteristics and self-reported pulmonary outcomes in Swiss childhood cancer survivors after hematopoietic stem cell transplantation—a cohort study

**DOI:** 10.1038/s41409-020-01137-1

**Published:** 2020-11-25

**Authors:** Maria Otth, Christina Schindera, Tayfun Güngör, Marc Ansari, Katrin Scheinemann, Fabiën N. Belle, Philipp Latzin, Nicolas von der Weid, Claudia E. Kuehni, for the Swiss Pediatric Oncology Group (SPOG), M. Ansari, M. Beck Popovic, J. P. Bourquin, P. Brazzola, J. Greiner, J. Rössler, F. Schilling, K. Scheinemann, N. von der Weid

**Affiliations:** 1 Childhood Cancer Research Group, Institute of Social and Preventive Medicine, University of Bern, Bern, Switzerland; 2 Division of Oncology-Hematology, Department of Pediatrics, Kantonsspital Aarau, Aarau, Switzerland; 3 Graduate School for Cellular and Biomedical Sciences, University of Bern, Bern, Switzerland; 4 Division of Hematology and Oncology, University Children’s Hospital Basel and University of Basel, Basel, Switzerland; 5 Division of Hematology/Oncology/Immunology and SCT, Children’s Research Center (CRC), University Children’s Hospital Zurich-Eleonore Foundation, Zurich, Switzerland; 6 Department of Women, Child and Adolescent, Oncology-Hematology Unit, Geneva University Hospital, Geneva, Switzerland; 7 CANSEARCH Research Laboratory, Medical Faculty, University of Geneva, Geneva, Switzerland; 8 University of Basel, Basel, Switzerland; 9 Department of Pediatrics, McMaster University Hamilton, Hamilton, ON Canada; 10 Center for Primary Care and Public Health (Unisanté), University of Lausanne, Lausanne, Switzerland; 11 Division of Respiratory Medicine, Department of Pediatrics, Inselspital, University Hospital, University of Bern, Bern, Switzerland; 12 Division of Pediatric Hematology/Oncology, Department of Pediatrics, Inselspital, Bern University Hospital, University of Bern, Bern, Switzerland; 13 Pediatric Hematology-Oncology Unit, Lausanne University Hospital, Centre Hospitalier Universitaire Vaudois, Lausanne, Switzerland; 14 Division of Oncology, University Children’s Hospital Zurich-Eleonore Foundation, Zurich, Switzerland; 15 Pediatria Bellinzona, Ospedale Regionale di Bellinzona e Valli, Bellinzona, Switzerland; 16 Division of Hematology and Oncology, Children’s Hospital of Eastern Switzerland, St. Gallen, Switzerland; 17 Division of Oncology/Hematology, Department of Pediatrics, Kantonsspital Luzern, Lucerne, Switzerland

**Keywords:** Paediatrics, Epidemiology, Risk factors

## Abstract

Childhood cancer survivors treated with hematopoietic stem cell transplantation are at high risk for pulmonary morbidity and mortality. In this retrospective study we described transplant characteristics of pediatric patients who underwent hematopoietic stem cell transplantation in Switzerland and how these characteristics changed over time, compared self-reported pulmonary outcomes between transplanted and non-transplanted survivors, and investigated risk factors for the reported pulmonary outcomes. As part of the population-based Swiss Childhood Cancer Survivor Study, we sent questionnaires to all ≥5-year childhood cancer survivors diagnosed 1976–2010 at age ≤20 years. We included 132 transplanted survivors and 368 matched non-transplanted survivors. During the study period transplant characteristics changed, with decreasing use of total body irradiation and increased use of peripheral blood stem cells and mismatched and unrelated donors as transplant source. One-fifth of transplanted survivors (20%, 95%CI 13–27%) and 18% of non-transplanted survivors (95%CI 13–21%) reported at least one pulmonary outcome. None of the analyzed factors was significantly associated with an increased risk of pulmonary outcomes in multivariable analysis. We found that pulmonary outcomes were frequently reported in transplanted and non-transplanted childhood cancer survivors, indicating a strong need for long-term pulmonary follow-up care.

## Introduction

Hematopoietic stem cell transplantation (HSCT) is an effective but intensive treatment for childhood cancer. HSCT can be performed as allogeneic or autologous transplantation and is used either as first line or salvage treatment [1–4]. The history of allogeneic HSCT goes back to the 1980 s and indications, conditioning regimens, and donor sources have changed enormously since then [5, 6]. Pulmonary damage and late effects due to lung toxic treatments and complications prior to, during, or after transplantation can occur in childhood cancer survivors (CCS) [7–9]. Lung toxic treatments include the chemotherapeutics bleomycin, busulfan, carmustine (BCNU) or lomustine (CCNU), radiation involving the lung tissue, total body irradiation (TBI), and thoracic surgery [10–13]. Transplant-specific pulmonary complications include idiopathic pulmonary syndrome and complications from the spectrum of pulmonary graft versus host disease (GvHD), such as bronchiolitis obliterans or bronchiolitis obliterans organizing pneumonia [7, 14–16]. Severe pulmonary infections are additional complications due to long-lasting neutropenic episodes.

Pulmonary symptoms and diseases, summarized as pulmonary outcomes, are associated with high morbidity in survivors [17–19]. Cohort studies showed that survivors more often report pulmonary outcomes than siblings [20, 21]. To date, pulmonary outcomes in survivors after HSCT have only been reported by few single-center studies [22, 23]. Data based on national population-based assessments of pulmonary outcomes are lacking.

This nationwide retrospective study describes transplant characteristics, such as transplant indications and conditioning regimens, of pediatric patients who had HSCT in Switzerland and how these characteristics changed over time. Then, we compared self-reported pulmonary outcomes between transplanted and non-transplanted survivors and investigated risk factors for reporting pulmonary outcomes.

## Methods

### The Swiss Childhood Cancer Survivor Study

The Swiss Childhood Cancer Survivor Study (SCCSS) is a long-term national cohort study of all patients registered in the Swiss Childhood Cancer Registry (SCCR) who have been diagnosed since 1976, have survived ≥5 years after initial diagnosis, and were alive at the time of study inclusion [24]. The SCCR is a nationwide, population-based cancer registry including all patients diagnosed below age <21 years with leukemia, lymphoma, central nervous system (CNS) tumors, malignant solid tumors, or Langerhans cell histiocytosis [25]. From 2007 to 2017, we sent questionnaires to parents of children aged 5–15 years, adolescents aged 16–19 years, and adult CCS aged ≥20 years. The Ethics Committee of the Canton of Bern approved the SCCR and SCCSS (KEK-BE: 166/2014). The SCCSS is registered at ClinicalTrials.gov (identifier: NCT03297034).

### Study population

We included all survivors who participated in the SCCSS and had been treated in a clinic affiliated to the Swiss Pediatric Oncology Group (SPOG) between 1976 and 2010. As the definition of 5-year survivors was based on the year of diagnosis and not the year of HSCT, some participants might have been transplanted <5 years before answering the questionnaire. As comparison group, we included survivors participating in the SCCSS who had not had a HSCT. Non-transplanted CCS were matched to transplanted CCS based on sex, diagnosis, age at diagnosis (range ± 2 years), and year of diagnosis (range ± 5 years) striving for a 1:3 ratio. Through this matching we wanted to achieve that CCS in both groups were as similar as possible in terms of sex, age at diagnosis and diagnosis, but did only differ by whether they had had HSCT or not. This matching did probably not eliminate all other differences between the groups since the reason for HSCT, such as high-risk status or relapse, already demands additional treatment for the HSCT patients.

### Treatment and transplant characteristics

We collected treatment- and transplant-related characteristics of transplanted survivors from medical records. We calculated cumulative doses for eight known or suspected lung toxic agents: bleomycin, busulfan, carmustin (BCNU), cyclophosphamide, ifosfamide, lomustin (CCNU), melphalan, and thiotepa [11–13, 26]. We combined cumulative doses of alkylating agents (all chemotherapeutics except of bleomycin) by calculating the cyclophosphamide equivalent dose (CED) [27]. We categorized the cumulative CED as either lower/equal to or higher than 11,300 mg/m^2^ with a median-split. We converted busulfan given orally to busulfan intravenously by multiplying it by factor 0.8 [28]. We categorized chest radiation as yes/no according to the Children’s Oncology Group guidelines version 5.0 [13] and included irradiation of the upper abdomen. We recorded surgery to the thorax, lung, chest wall, mediastinum, and thoracic spine. Needle biopsies and implantation of venous devices were not coded as thoracic surgery. We collected date of transplantation, history of relapse, remission status, source of transplant, stem cell donor, cytomegalovirus (CMV) status, sex and blood group of donor and recipient, and information on graft versus host disease (GvHD). We categorized stem cell transplantation into autologous and allogeneic and further specified allogeneic transplantation into Human Leucocyte Antigen (HLA) matched (e.g., 12/12) and HLA-mismatched (e.g., 9/10) donors. As HLA typing and documentation changed substantially in the last decades, it was not possible to assess exact HLA matching [29, 30]. We categorized GvHD into acute and chronic according to information from medical records.

### Pulmonary outcomes

We collected information on pulmonary symptoms (chronic cough defined as ≥3 months) and diseases (pneumonia in last 2 years, lung fibrosis, emphysema, or chest wall abnormality) from the SCCSS questionnaires. We categorized the different pulmonary outcomes as yes/no (present/absent) variables. In addition to answering the questions, participants could describe other problems as free text. Responses we could not assign to one of the existing categories were coded as “other pulmonary problem”. We had ≤5% missings on pulmonary outcomes except for pneumonia (6% missings). We allocated missing information to “not having the pulmonary outcome” assuming that survivors would mention pulmonary outcomes if they were clinically significant.

### Clinical and lifestyle characteristics

We extracted the following clinical characteristics from the SCCR: sex, age at diagnosis, year of diagnosis, and cancer diagnoses according to the International Classification of Childhood Cancer, 3^rd^ edition [31]. For analyses, we used the following four diagnostic categories: leukemia, where patients with relapsed and refractory disease often receive allogeneic HSCT, lymphoma, and neuroblastoma, where autologous HSCT is used for relapsed or high-risk disease, and other diagnoses, where HSCT is used less frequently. For lifestyle characteristics we extracted smoking status from the questionnaires (Supplementary Explanation E1).

### Statistical analysis

We used descriptive statistics to describe sociodemographic, lifestyle, and clinical characteristics of transplanted and non-transplanted CCS. To assess trends in transplant characteristics across transplant eras we used the “nptrend” command in STATA software [32]. We compared the prevalence of pulmonary outcomes between transplanted and non-transplanted CCS using chi-square tests. We used logistic regression and likelihood ratio tests to quantify associations between sociodemographic, lifestyle, clinical, and transplant-related variables and pulmonary outcomes in transplanted CCS. We retained variables with a *p* value ≤ 0.1 in the univariable analysis for inclusion into the multivariable model and included radiotherapy as a priori confounder according to the literature. We compared sociodemographic and clinical characteristics of transplanted CCS who did or did not respond to the questionnaire by using chi-square tests and student’s *t*-tests. We used STATA software (Version 16.0, Stata Corporation, Austin, TX) to analyze the data.

## Results

### Characteristics of study population

We included 132 transplanted and 368 matched non-transplanted CCS (Supplementary Figs. F1 and F2). Transplanted responders and non-responders did not differ in sociodemographic, lifestyle, and clinical characteristics (Supplementary Table S1). The median age of transplanted CCS was 6.5 years (interquartile range, IQR 2.9–11.6 years) at cancer diagnosis and 8.8 years (IQR 4.8–13.6) at transplantation. Median follow-up time was 9.8 years (IQR 7.2–15.9). Leukemia was the most frequent cancer diagnosis (55%), followed by lymphoma (15%), and neuroblastoma (14%) (Table 1).

**Table 1:** Characteristics of transplanted (*N* = 132) and non-transplanted (*N* = 368) childhood cancer survivors, matched by sex, age at diagnosis, diagnosis, and year of diagnosis (1:3 ratio).

	Transplanted CCS (*n* = 132)	Non-transplanted CCS (*n* = 368)
	*n* (%)	*n* (%)
**Sociodemographic and lifestyle characteristics**		
Sex, male	69 (52)	195 (53)
Age at questionnaire, median years (IQR)	18.4 (13.8–22.9)	18.5 (13.6–23.8)
Smoking status^a^		
Active smoking	7 (5)	27 (7)
Passive smoking	63 (48)	163 (44)
Former active smoking	9 (6)	20 (6)
Never smoking	54 (41)	158 (43)
**Clinical characteristics**		
Age at diagnosis, median years (IQR)	6.5 (2.9–11.6)	6.4 (2.7–11.4)
Age at transplantation, median years (IQR)	8.8 (4.8–13.6)	NA
Follow-up time^b^, median years (IQR)	9.8 (7.2–15.9)	10.1 (7.9–15.1)
**Era of diagnosis**		
1976–1995	40 (30)	120 (33)
1996–2005	60 (45)	156 (42)
2006–2010	32 (25)	92 (25)
**Childhood cancer diagnosis according to ICCC-3**		
I: Leukemia	72 (55)	214 (58)
II: Lymphoma	20 (15)	60 (16)
IV: Neuroblastoma	19 (14)	44 (12)
Other^c^	21 (16)	50 (14)

*CCS* childhood cancer survivors, *ICCC-3* International Classification of Childhood Cancer, 3rd edition, *IQR* interquartile range.

^a^“Active” and “former active smoking” assessed in adolescents and adults; “passive smoking” in children corresponds to having parents who currently smoke or formerly smoked, “never smoking” in children corresponds to having both parents who never smoked.

^b^Time from first diagnosis until date of answering the questionnaire.

^c^Other tumors in transplanted survivors include: tumors of the central nervous system (*n* = 6), retinoblastoma (*n* = 1), malignant bone tumors *(n* = 7), soft tissue sarcomas (*n* = 4), malignant germ cell tumors (*n* = 3) Other tumors in non-transplanted survivors include: tumors of the central nervous system (*n* = 12), retinoblastoma (*n* = 1), malignant bone tumors (*n* = 9), soft tissue sarcomas (*n* = 4), malignant germ cell tumors (*n* = 3).

### Transplant characteristics and change over time

The absolute number of transplanted CCS who participated in the SCCSS increased over time. Leukemia remained the most common underlying cancer diagnosis in all three eras (Table 2). Conditioning regimens changed with a relative but non-significant reduction in TBI-containing regimens from 61% in the first to 39% in the other two eras (*p* for trend = 0.083). Among chemotherapeutics, the proportion of CCS who received ifosfamide increased (*p* = 0.002) but the median cumulative dose decreased non-significantly (*p* = 0.477). Also cyclophosphamide dosage decreased (*p* < 0.001) with no significant reduction in the proportion of CCS receiving it (*p* = 0.186). For bleomycin there was a trend towards lower cumulative doses in more recent eras (*p* = 0.094). Two-thirds (65%) of CCS had radiotherapy involving the thorax with no significant change over time, and 9% had thoracic surgery with a trend to an increasing proportion of CCS in more recent years. Nearly half of transplanted CCS received autologous HSCT (46%) and in 57% HSCT was performed in first remission or refractory disease. The proportion of transplanted CCS receiving peripheral blood stem cells increased from 27% to 71% with a corresponding reduction in the proportion of those receiving bone marrow stem cells (*p* for trend <0.001). Eight CCS developed chronic GvHD (cGvHD) but none had pulmonary GvHD (Supplementary Table S2). Supplementary Tables S2 and S3 provide summaries of clinical, treatment, and transplant characteristics for CCS transplanted in autologous or allogeneic settings, stratified by era of transplantation. Differences in CCS exposed to allogeneic or autologous HSCT are shown in Supplementary Table S4.

**Table 2:** Characteristics of transplanted childhood cancer survivors (*N* = 132) stratified by era of transplantation.

	Total (*n* = 132)	1976–1995 (*n* = 33)	1996–2005 (*n* = 51)	2006–2015 (*n* = 48)	*p* value*
	*n* (%)	*n* (%)	*n* (%)	*n* (%)	
**Clinical characteristics**					
Cancer diagnosis according to ICCC-3					0.806
I: Leukemia	72 (55)	18 (55)	26 (51)	28 (58)	
II: Lymphoma	20 (15)	8 (24)	7 (14)	5 (10)	
IV: Neuroblastoma	19 (14)	5 (15)	8 (16)	6 (13)	
Other^a^	21 (16)	2 (6)	10 (19)	9 (19)	
**Treatment characteristics**					
Conditioning containing TBI	59 (45)	20 (61)	20 (39)	19 (39)	0.083
Conditioning regimens					0.003
TBI + cyclophosphamide ± others	34 (26)	16 (48)	12 (23)	6 (13)	
TBI + others	25 (19)	4 (12)	8 (16)	13 (27)	
Busulfan + cyclophosphamide ± other	28 (21)	7 (21)	11 (21)	10 (21)	
Busulfan ± others	8 (6)	1 (3)	4 (8)	3 (6)	
Carmustine ± others	9 (7)	3 (9)	3 (6)	3 (6)	
Cyclophosphamide ± others	9 (7)	1 (3)	6 (12)	2 (4)	
Melphalan ± carboplatin ± others	19 (14)	1 (3)	7 (14)	11 (23)	
Chemotherapeutic agents					
Alkylating agents combined^b^	131 (99)	33 (100)	50 (98)	48 (100)	
Busulfan	37 (28)	9 (27)	16 (31)	12 (25)	0.776
Carmustine	9 (7)	4 (12)	3 (6)	2 (4)	0.180
Cyclophosphamide	123 (93)	33 (100)	46 (90)	44 (92)	0.186
Ifosfamide	62 (47)	9 (27)	23 (45)	30 (63)	0.002
Lomustine	2 (2)	1 (3)	-	1 (2)	0.835
Melphalan	44 (33)	9 (27)	17 (33)	18 (38)	0.342
Thiotepa	14 (11)	3 (9)	8 (16)	3 (6)	0.563
Bleomycin	8 (6)	3 (9)	3 (6)	2 (4)	0.371
Chemotherapeutic agents, mg/m^2^ (IQR)					
Alkylating agents combined^b^	11329 (5687–17164)	11658 (7924–17391)	11367 (5879–21425)	8546 (4447–16131)	0.199
Busulfan	443 (324–480)	480 (470–587)	344 (297–480)	440 (374–449)	0.021
Carmustine	300 (298–300)	300 (298–351)	300 (298–300)	300 (291–306)	0.737
Cyclophosphamide	4200 (3021–7535)	7299 (4200–8684)	4247 (3090–8230)	3439 (2634–5258)	<0.001
Ifosfamide	9941 (4032–22500)	11500 (5200–16032)	10227 (4032–22500)	8181 (4017–19767)	0.477
Lomustine	395 (190–600)	190	–	600	0.317
Melphalan	140 (139–169)	140 (140–142)	140 (140–140)	140 (139–180)	0.739
Thiotepa	680 (588–900)	750 (168–900)	749 (591–900)	610 (307–900)	0.921
Bleomycin	40 (40–46)	42 (40–80)	40 (40–50)	30 (20–40)	0.094
Radiotherapy involving the thorax^c^	86 (65)	25 (76)	32 (63)	29 (60)	0.175
Thoracic surgery^d^	12 (9)	1 (3)	5 (10)	6 (13)	0.157
**Transplant characteristics**					
Remission status at transplantation					0.906
First remission	75 (57)	16 (48)	35 (69)	24 (50)	
Relapsed disease	57 (43)	17 (52)	16 (31)	24 (50)	
Stem cell donor					0.098
Autologous	61 (46)	17 (52)	25 (49)	19 (39)	
HLA identical sibling / HLA matched (un-)relative donor	56 (42)	16 (48)	19 (37)	21 (44)	
HLA mismatch (un-)related /haploidentical	15 (11)	0	7 (14)	8 (17)	
Source of transplant					<0.001
Cord blood	6 (5)	–	1 (2)	5 (10)	
Peripheral blood	75 (57)	9 (27)	32 (63)	34 (71)	
Bone marrow	46 (35)	22 (67)	17 (33)	7 (15)	
Unknown	5 (4)	2 (6)	1 (2)	2 (4)	
Pulmonary outcome	26 (20)	7 (21)	11 (22)	8 (17)	0.582

*HLA* human leukocyte antigen, *ICCC-3* International Classification of Childhood Cancer, 3rd edition, *IQR* interquartile range, *N* number, *TBI* total body irradiation.

*p value for trend.

^a^Other tumors include: tumors of the central nervous system (*n* = 6), retinoblastoma (*n* = 1), malignant bone tumors (*n* = 7), soft tissue sarcoma (*n* = 4), malignant germ cell tumors (*n* = 3).

^b^Combination according to Cyclophosphamide Equivalent Dose (CED) [27].

^c^Thoracic radiation fields according to COG guidelines, Version 4.0, Oct 2018, including radiation to the chest, whole lung, mediastinum, (mini-)mantle field, TBI and additionally upper abdomen and thoracic spine, including craniospinal irradiation.

^d^Thoracic surgery according to COG guidelines, Version 4.0, Oct 2018, including thoracotomy, chest wall surgery, rib resection, lobectomy, pulmonary metastasectomy and wedge resection.

### Prevalence of pulmonary outcomes

**Fig. 1:**
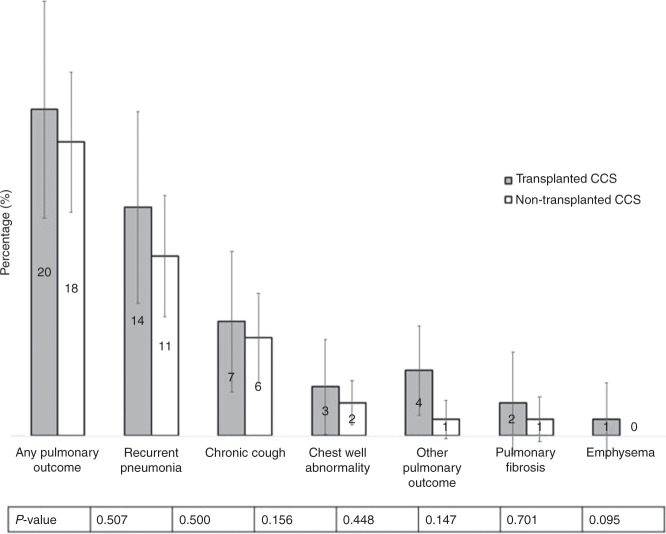
Prevalence of self-reported pulmonary diseases and symptoms in transplanted (*N* = 132) and non-transplanted matched childhood cancer survivors (*N* = 368). Error bars represent 95% confidence intervals. *P* value comparing prevalence between transplanted and non-transplanted survivors. *Total N reduced for pulmonary fibrosis and emphysema because question only asked in adolescents and adults: *N* = 85 transplanted survivors, *N* = 195 non-transplanted survivors. ** “Other pulmonary outcome” includes reduced lung function (*n* = 3) and pulmonary GvHD (*n* = 1).

Any pulmonary outcome was reported as often in transplanted (20%) as in non-transplanted CCS (18%; *p* = 0.507). The occurrence of the listed pulmonary outcomes was not significantly different between transplanted and non-transplanted CCS. Pneumonia was the most frequently reported outcome (Fig. 1). The proportion of transplanted CCS reporting any pulmonary outcome did not change by era of transplantation (Table 2).

### Risk factors for pulmonary outcomes

In univariable logistic regression analysis, we found associations between older age at cancer diagnosis (increase per year; odds ratio [OR] 1.2, 95% confidence interval [CI] 1.05–1.28), exposure to bleomycin (OR 4.63, 95%CI 1.08–19.97), and thoracic surgery (OR 7.44, 95%CI 2.13–25.92) with any pulmonary outcome (Table 3). We found no significant association with era of diagnosis, treatment with other chemotherapeutics, median CED, and thoracic radiotherapy, but numbers were small. Transplant-related factors were also not significantly associated with reporting of pulmonary outcomes (Table 3). In multivariable logistic regression analysis, the effect of thoracic surgery was reduced to an OR of 3.91 (95%CI 0.95–16.02), suggesting that it has been confounded by other factors related to disease and treatment (Table 4). Most of the 12 CCS treated with thoracic surgery have been diagnosed with Ewing sarcoma (*n* = 7) or lymphoma (*n* = 4). All except one CCS have been transplanted autologous, most suffered from relapsed disease (*n* = 10), received radiotherapy to the chest (*n* = 10), were treated with open thoracic surgeries (*n* = 9), received at least one lung toxic chemotherapeutic agent (*n* = 7), or have been exposed to a combination of radiotherapy and thoracic surgery or resection of lung tissue (*n* = 9) (Supplementary Table S5).

**Table 3:** Association between sociodemographic, clinical, treatment, and transplant characteristics on self-reported pulmonary outcomes.

	Reporting of any pulmonary outcome					
	*n*_outcome_	*N*_total_	%	OR	95% CI	*p* value*
**Sociodemographic and lifestyle characteristics**						
Sex						0.795
Male	13	69	19	1		
Female	13	63	21	1.12	0.47–2.64	
Age at questionnaire, continuous (years)	26	132	20	1.05	0.99–1.11	0.088
Smoking status^a^						0.924
Never smoking	12	60	20	1		
Passive smoking	10	56	18	0.87	0.34–2.21	
Former active smoking	2	9	22	1.14	0.21–6.21	
Active smoking	2	7	29	1.60	0.27–9.28	
**Clinical characteristics**						
Age at diagnosis, continuous (years)	26	132	20	1.2	1.05–1.28	0.002
Follow-up time, continuous (years)	26	132	20	0.98	0.91–1.06	0.613
Era of diagnosis						0.800
1976–1990	5	20	25	1		
1991–2000	8	45	18	0.64	0.18–2.31	
2001–2010	13	67	19	0.72	0.22–2.35	
Cancer diagnosis according to ICCC-3						0.271
Leukemia	15	72	21	1		
Lymphoma	5	20	25	1.27	0.39–4.04	
Neuroblastoma	1	19	5	0.21	0.03–1.71	
Other^b^	5	21	24	1.18	0.37–3.76	
**Treatment characteristics**						
Bleomycin						0.040
No	22	124	18	1		
Yes	4	8	50	4.63	1.08–19.97	
Busulfan						0.190
No	16	95	17	1		
Yes	10	37	27	1.83	0.74–4.51	
Nitrosureas (BCNU and CCNU)						0.107
No	22	122	18	1		
Yes	4	10	40	3.03	0.79–11.65	
Cyclophosphamide						0.844
No	2	9	22	1		
Yes	24	123	20	0.84	0.16–4.34	
Ifosfamide						0.596
No	15	70	21	1		
Yes	11	62	18	0.79	0.33–1.88	
Melphalan						0.281
No	15	88	17	1		
Yes	11	44	25	1.62	0.67–3.91	
Treosulfan						0.400
No	24	126	19	1		
Yes	2	6	33	2.12	0.36–12.28	
Alkylating agents^c^						0.084
≤11,300 mg/m^2^	9	66	14	1		
>11,300 mg/m^2^	17	66	26	2.19	0.89–5.37	
Radiotherapy to chest (including TBI)						0.165
No	6	46	13	1		
Yes	20	86	23	2.02	0.75–5.45	
Thoracic surgery						<0.001
No	19	120	16	1		
Yes	7	12	58	7.44	2.13–25.92	
**Transplant characteristics**						
Remission status at transplantation						0.223
First remission/primary refractory	12	75	16	1		
Relapsed disease	14	57	24	1.71	0.72–4.05	
Type of transplantation						0.995
Allogeneic	14	71	20	1		
Autologous	12	61	20	0.99	0.42–2.35	
Stem cell donor						0.739
Autologous	12	61	20	1		
HLA ident. sibling, matched (un)related donor	11	56	20	0.99	0.40–2.48	
HLA mismatched (un)related, haploidentical	3	15	20	1.02	0.25–4.19	
Source of transplant (*n* = 66)^d^						0.933
Bone marrow	7	34	21	1		
Peripheral blood	6	26	23	1.15	0.34–3.98	
Cord blood	1	6	17	0.77	0.08–7.71	
Graft versus host disease (*n* = 71)^d^						0.449
No	4	15	27	1		
Yes	10	56	18	0.59	0.16–2.27	

Results from univariable logistic regression analysis. *N* = 132, median age at study 18.4 years.

*BCNU* Lomustine, *CCNU* Carmustine, *HLA* human leukocyte antigen, *ICCC-3* International Classification of Childhood Cancer, 3rd edition, *OR* Odds ratio, *TBI* total body irradiation, *CI* confidence interval.

*p value calculate by logistic regression (Wald test) for continuous and binary independent variables and by likelihood ratio test for independent variables with >2 categories.

^a^Active and former active smoking assessed in adolescents and adults. Passive and never smoking assessed in children, adolescent and adults.

^b^Other diagnostic groups include: malignant bone tumors (*n* = 7), tumors of the central nervous system (*n* = 6), soft tissue sarcomas (*n* = 4), germ cell tumors (*n* = 3), retinoblastoma (*n* = 1).

^c^Cumulative alkylating dose according to cyclophosphamide equivalent dose (CED); categorized in smaller or equal to the median or larger as the median cumulative dose.

^d^In survivors undergone allogeneic transplantation only.

**Table 4:** Association between sociodemographic, clinical, treatment, and transplant characteristics on self-reported pulmonary outcomes.

	OR	95% CI	*p* value
Age at diagnosis	1.13	0.99–1.28	0.055
Age at questionnaire	1.00	0.91–1.08	0.892
Bleomycin exposure	1.57	0.28–8.81	0.608
Median CED dose >11,300 mg/m^2^	1.74	0.57–5.33	0.330
Thoracic surgery	3.91	0.95–16.02	0.058
Thoracic radiotherapy	1.58	0.49–5.14	0.446

Results from multivariable logistic regression analysis, adjusted for all factors in the table. *N* = 132, median 18.4 years at study.

*CED* cyclophosphamide equivalent dose, *CI* confidence interval, *OR* Odds ratio.

## Discussion

This nationwide population-based cohort study found that transplant characteristics changed over time with fewer HSCT recipients receiving TBI or lung toxic chemotherapeutics. One-fifth of ≥5-year CCS reported at least one pulmonary outcome 10 years after cancer diagnosis irrespective of whether they had been transplanted or not. Our analyses point to older age at diagnosis and thoracic surgery as possible risk factors for self-reported pulmonary outcomes.

TBI is a crucial component of conditioning regimens for allogeneic HSCT, but known to be lung toxic. Even though TBI cannot completely be replaced by chemotherapy, such as in acute lymphoblastic leukemia [33], we found that the use of TBI has become less common in more recent eras. There was a non-significant trend towards lower cumulative doses of bleomycin and we found no evidence for a change in cumulative doses of carmustine, but numbers were small. The increasing use of peripheral blood stem cells in more recent eras is in line with literature [34–36] and the increasing use of mismatched (un-) related donors reflects the overall progress in HSCT over time.

The proportion of transplanted CCS reporting any pulmonary outcome did not change during the three HSCT eras. Studies that compared self-reported pulmonary outcomes in transplanted CCS are few. Fanfulla et al. examined children during the first 18 months after allogeneic HSCT [22]. Cough was reported by 15–25% of children and pneumonia was diagnosed in the first 6 months in 19% of children. The occurrence of pneumonia in the first 6 months, is indicative of delayed immune reconstitution rather than late pulmonary outcomes. Since the follow-up (18 months) is shorter than in our population (10 years) direct comparison is difficult. Also in the entire cohort of Swiss CCS (*N* = 1 894) pneumonia was the most frequently reported pulmonary outcome (10%), and pulmonary fibrosis (0.8%) and emphysema (0.2%) were reported by few CCS [20]. CCS in the North American Childhood Cancer Survivor Study showed a different distribution of pulmonary outcomes with chronic cough being the most frequent outcome (7.8%), followed by pulmonary fibrosis (1.9%), and recurrent pneumonia (1.7%) [21]. We found no difference in the prevalence of pulmonary outcomes between transplanted and non-transplanted CCS in our study (20% vs. 18%). This could be explained by the high proportion of leukemia (58%) and lymphoma (16%) diagnoses in non-transplanted CCS due to the matching. A Danish cohort study included 94 leukemia survivors a median of 10 years from diagnosis, treated with chemotherapy only, and 11% suffered from pulmonary problems, mainly cough [37]. A US study including Hodgkin’s lymphoma survivors treated with chest radiation but without HSCT showed that 17% had at least one episode of pneumonia and 9% reported dyspnea [38].

CCS who had undergone thoracic surgery in addition to HSCT reported more pulmonary outcomes than those without thoracic surgery. This might be because this group of CCS had received more often thoracic radiotherapy or lung toxic chemotherapeutics, had more often been diagnosed with relapsed disease, and underwent open thoracic surgeries in most cases, which goes along with a more intensive treatment. Residual confounding by these additional lung toxic treatment modalities probably leads to an overestimation of the association between thoracic surgery and pulmonary outcomes. Older age at diagnosis, resulting in older age at HSCT, was another risk factor for pulmonary outcomes in univariable analysis. No study has assessed self-reported pulmonary outcomes in the context of age at HSCT, but four studies showed an association between older age at HSCT and deterioration in selected pulmonary function parameters [23, 39–41]. In multivariable analysis, bleomycin was not a risk factor for pulmonary outcomes anymore, which is in line with findings from the whole Swiss CCS cohort [20]. In our cohort, we found no significant effect of other selected chemotherapeutics and transplant-related factors on the reporting of pulmonary outcomes. All studies that evaluated the impact of cGvHD on the lung, used pulmonary function tests as outcome measure [41–44]. They reported a negative effect of cGvHD on pulmonary function. We explain the missing effect of cGvHD, lomustine, and carmustine by the low number of survivors exposed to each of these factors. Also some CCS with severe pulmonary cGvHD might have died before receiving the SCCSS questionnaire and missing or non-detailed documentation in the medical records might have led to an underestimation of the effect of cGvHD on pulmonary outcomes.

We found no difference in pulmonary outcomes between CCS treated with autologous and allogeneic HSCT. Thoracic surgery was overrepresented in the autologous group because of the underlying diagnoses, mainly bone tumors. In contrast, CCS treated with allogeneic HSCT were more often exposed to chest radiotherapy, which can lead to radiation pneumonitis and an increased risk of interstitial pneumonitis due to infections such as CMV. Both factors have not been assessed in detail.

The strengths of this study include the population-based national design of the SCCSS, the high response rate of transplanted CCS (71%), and the comparability between responding and nonresponding transplanted survivors. This makes us confident, that our results can be extrapolated to ≥5-year Swiss survivors who underwent HSCT. In addition, the completeness of exact treatment exposure, including cumulative doses of chemotherapeutics and detailed information on HSCT in transplanted CCS is another strength.

The reliance on self-reported outcome data is a limitation and our study did not include objective pulmonary function tests. CCS treated with open thoracic surgery are reminded by the scar of their history and thus may be more sensitive in dealing with their lung health, and may remember and report pulmonary outcomes better. However, Louie et al. reported a high agreement between self-reported pulmonary outcomes, such as chronic cough, pulmonary fibrosis, and emphysema, and their validation by extractions from medical records (sensitivity 96.2%; specificity 90.8%) [45]. The SCCSS has not been designed for survivors after HSCT specifically, neither for the assessment of pulmonary outcomes only and did not include specific questions on exertion-induced dyspnea or effort intolerance. This might have led to underreporting of pulmonary outcomes in our study. Also “pneumonia” might have been misunderstood by lay persons, as it was not defined in the questionnaire. Survival bias due to inclusion of ≥5-year survivors could have led to underestimation of pulmonary outcomes as more severely affected patients might have died. The small number of transplanted CCS who have been exposed to specific chemotherapeutics and transplant-related exposures did not allow for a multivariable analysis of all exposures in a single model. Also the detailed information on treatment exposures, such as cumulative doses, was only available for transplanted CCS. Finally, the absolute numbers of CCS reporting pulmonary outcomes was small, because the study population was young with a relatively short follow-up time, and the incidence of pulmonary outcomes increases over lifetime [20, 21, 39, 46].

In summary, we found that one-fifth of CCS, including those who underwent HSCT and matched controls, developed long-term pulmonary outcomes. As we only assessed self-reported outcomes, using a limited number of questions, this proportion probably only represents the tip of the iceberg. This underlines that we should implement long-term pulmonary follow-up recommendations on a large scale [13, 47–49] using sensitive outcome measures, such as lung function tests, to assess the full spectrum of long-term pulmonary sequelae after childhood cancer at an early stage.

## Supplementary information

Supplemental material
