# Supplementary material for: Transplant characteristics and self-reported pulmonary outcomes in Swiss childhood cancer survivors after hematopoietic stem cell transplantation—a cohort study
Source: Bone Marrow Transplant. 2020 Nov 25;56(5):1065–76. doi: 10.1038/s41409-020-01137-1 (PMC8113058; doi:10.1038/s41409-020-01137-1)
Supplement: Supplementary file 1 — Supplemental material [file 41409_2020_1137_MOESM1_ESM.docx]

**Online Supplement**

# **Transplant characteristics and self-reported pulmonary outcomes in Swiss childhood cancer survivors after hematopoietic stem cell transplantation – a cohort study**

Maria Otth, MD, Christina Schindera, MD, Tayfun Güngör, MD, Marc Ansari, MD, Katrin Scheinemann, MD, Fabiën N. Belle, PhD, Philipp Latzin, MD PhD, Nicolas von der Weid^,^ MD, Claudia E. Kuehni, MD, for the Swiss Pediatric Oncology Group (SPOG)

**SUPPLEMENTAL EXPLANATON E1**

This supplemental document describes how we categorized smoking status in childhood cancer survivors participating in the Swiss Childhood Cancer Survivor Study

**Lifestyle variables**

We combined the answers on active and passive smoking into one variable with four categories: “active smoking”, “passive smoking”, “former active smoking”, and “never active smoking”. The category “passive smoking” includes adolescent and adult CCS who state that they are exposed to passive smoking and minor CCS, whose parents currently smoke or are former smokers. The category “never smoking” includes adolescents and adults, who never smoked and were not exposed to passive smoking, and children, whose parents never smoked.

*Smoking questions asked in the adult and adolescent version*

Do you currently smoke cigarettes?

No, I never smoked

No, I stopped smoking since ___ month

Yes, I smoke irregularly: number ___ of cigarettes per week

Yes, I smoke regularly: number ___ of cigarettes per day

If you count up all the situations, how many hours per day are you normally exposed to

tobacco smoke of other people?

___ hours

*Smoking questions asked in parents’ version (separate for mother and father)*

Have you ever smoked?

No, never

Yes, stopped since _____

Yes, still smoke today

**SUPPLEMENTAL TABLE S1**:

This supplemental table describes the characteristics of transplanted childhood cancer survivors eligible for the questionnaire stratified into responder and non-responder to the questionnaire, N=200. The patient tree is described in **Supplemental Figure F2**.

|  | **Responder**  **(n=132)** | **Non-responder**  **(n=68)** | **p value^1^** |
| --- | --- | --- | --- |
|  | n (%) | n (%) |  |
| **Sociodemographic characteristics** |  |  |  |
| **Sex**, male | 69 (52) | 43 (63) | 0.139 |
| **Age at survey**, median years (IQR) | 18.4 (13.8 – 22.9) | 18.6 (14.3 – 22.6)^2^ | 0.933 |
| **Language region**  German  French or Italian | 89 (67)  43 (33) | 46 (68)  22 (32) | 0.975 |
| **Clinical characteristics** |  |  |  |
| **Age at diagnosis,** median years (IQR) | 6.5 (2.9 - 11.7) | 6.7 (2.8, 11.8) | 0.876 |
| **Age at first HSCT,** median years (IQR) | 8.8 (4.8 - 13.6) | 11.1 (4.7 – 14.1) | 0.221 |
| **Follow-up time ^2^**, median years (IQR) | 9.8 (7.2 - 15.9) | 10.8 (7.6 – 14.9) | 0.978 |
| **Era of diagnosis**  1976-1995  1996-2005  2006-2010 | 40 (30)  60 (46)  32 (24) | 22 (32)  33 (49)  13 (19) | 0.712 |
| **Cancer diagnosis according to ICCC-3**  I: Leukemia  Ia: lymphoid leukemia  Ib: acute myeloid leukemia  Ic-e: CML, MDS, unspecified other  leukemias  II: Lymphoma  IIa: Hodgkin lymphoma  IIb: Non-Hodgkin lymphoma  IIc: Burkitt lymphoma  IId: miscellaneous  IV: Neuroblastoma  Other^5^ | 72 (55)  43 (33)  18 (14)  11 (8)  20 (15)  7 (5)  8 (6)  4 (3)  1 (1)  19 (14)  21 (16) | 32 (46)  21 (31)  8 (11)  3 (4)  14 (21)  1 (2)  7 (10)  6 (9)  -  8 (12)  14 (21) | 0.561 |
| **History of any relapse**  Yes  No | 62 (47)  70 (53) | 42 (62)  26 (38) | 0.047 |

HSCT, hematopoietic stem cell transplantation; ICCC-3, International Classification of Childhood Cancer, 3^rd^ edition; IQR, interquartile range; N, number

^1^ chi-squared for categorical variables; t-test for continuous variables

^2^ Time from first diagnosis until answering the survey (responder) or sending the survey (nonresponder)

^3^ Other diagnosis in nonresponder include: tumors of the central nervous system (n=6), retinoblastoma (n=1),

renal tumors (n=1), hepatic tumors (n=1), malignant bone tumors (n=3), soft tissue sarcoma (n=1), malignant

germ cell tumors (n=1)

Other diagnosis in responder include: tumors of the central nervous system (n=8), retinoblastoma (n=1),

malignant bone tumors (n=7), soft tissue sarcomas (n=4), malignant germ cell tumors (n=1)

**SUPPLEMENTAL TABLE S2:**

This supplemental table describes clinical, treatment and transplant characteristics of childhood cancer survivors transplanted allogeneic (N=71) stratified by era of transplantation

|  | **1976-1995**  (n=16) | **1996-2005**  (n=26) | **2006-2015**  (n=29) | **Total**  (n=71) |
| --- | --- | --- | --- | --- |
|  | n (%) | n (%) | n (%) | n (%) |
| **Clinical characteristics** |  |  |  |  |
| **Cancer diagnosis according to ICCC-3**  I: Leukemia  II: Lymphoma  IV: Neuroblastoma  Other^1^ | 16 (100)  0  0  0 | 23 (88)  3 (12)  0  0 | 29 (100)  0  0  0 | 68 (96)  3 (4)  0  0 |
|  |  |  |  |  |
| **Treatment characteristics** |  |  |  |  |
| **Conditioning containing TBI** | 15 (94) | 17 (65) | 18 (62) | 50 (70) |
| **Conditioning regimens** |  |  |  |  |
| TBI + cyclophosphamide ± others | 13 (81) | 12 (46) | 6 (21) | 31 (44) |
| TBI + others | 2 (13) | 5 (19) | 12 (42) | 19 (27) |
| Busulfan + cyclophosphamide ± other | 1 (6) | 9 (35) | 9 (31) | 19 (27) |
| Busulfan ± others | - | - | 1 (3) | 1 (1) |
| Melphalan ± Carboplatin ± others | - | - | 1 (3) | 1 (1) |
| **Chemotherapeutic agents** |  |  |  |  |
| Alkylating agents combined ^2^ | 16 (100) | 25 (96) | 29 (100) | 70 (99) |
| Busulfan | 2 (13) | 9 (35) | 9 (31) | 20 (28) |
| Carmustine | - | - | - | - |
| Cyclophosphamide | 16 (100) | 25 (96) | 28 (97) | 69 (97) |
| Ifosfamide | 4 (25) | 8 (31) | 14 (48) | 26 (37) |
| Lomustin | - | - | - | - |
| Melphalan | - | 4 (15) | 3 (10) | 7 (10) |
| Thiotepa | 1 (6) | 1 (4) | 1 (4) | 3 (4) |
| Bleomycin | 1 (6) | - | - | 1 (1) |
| **Chemotherapeutic agents, mg/m^2^** |  |  |  |  |
| Alkylating agents combined ^2^ | 7881 (4034 – 9588) | 5892 (4000 – 10631) | 5881 (3791 – 8006) | 6100 (3871 – 9579) |
| Busulfan | 520 (454 – 587) | 320 (297 – 345) | 431 (324 – 449) | 360 (320 – 445) |
| Carmustine | - | - | - | - |
| Cyclophosphamide | 5540 (3767 – 7881) | 3738 (2983 – 5372) | 3439 (2927 – 5570) | 3990 (2983 – 6242) |
| Ifosfamide | 7600 (4600 – 16948) | 4003 (3052 – 6016) | 4002 (2410 – 5923) | 4011 (3879 – 6126) |
| Lomustine | - | - | - | - |
| Melphalan | - | 140 (139 – 140) | 140 (138 – 140) | 140 (139 – 140) |
| Thiotepa | 168 | 304 | 307 | 304 (168 – 307) |
| Bleomycin | 80 | - | - | 80 |
| **Radiotherapy involving the thorax ^3^** | 15 (94) | 18 (69) | 18 (62) | 51 (72) |
| **Thoracic surgery ^4^** | 0 (0) | 1 (4) | 0 (0) | 1 (1) |
| **Transplant characteristics** |  |  |  |  |
| **Remission status at transplantation**  First remission  Relapsed disease | 8 (50)  8 (50) | 17 (65)  9 (35) | 13 (45)  16 (55) | 38 (54)  33 (46) |
| **Stem cell donor** |  |  |  |  |
| HLA identical sibling / HLA matched other relative | 13 (81) | 15 (58) | 11 (38) | 39 (55) |
| HLA matched unrelated | 3 (19) | 4 (15) | 10 (34) | 17 (24) |
| HLA mismatch related / haploidentical | - | 5 (19) | 2 (7) | 7 (10) |
| HLA mismatch unrelated | - | 2 (8) | 6 (21) | 8 (11) |
| **Source of transplant** |  |  |  |  |
| Cord blood | - | 1 (4) | 5 (17) | 6 (8) |
| Peripheral blood | 1 (6) | 10 (38) | 15 (52) | 26 (37) |
| Bone marrow | 13 (81) | 14 (54) | 7 (24) | 34 (48) |
| Unknown | 2 (13) | 1 (4) | 2 (7) | 5 (7) |
| **CMV status**  Donor and recipient IgG negative  Donor and recipient IgG positive  Donor and recipient IgG mismatch  Donor or recipient missing | 7 (44)  2 (12)  4 (25)  3 (19) | 12 (46)  7 (27)  5 (19)  2 (8) | 14 (48)  5 (17)  10 (35)  - | 33 (46)  14 (19)  19 (26)  5 (9) |
| **Sex match recipient/donor ^6^**  Match  Mismatch  Missing | 10 (62)  4 (25)  2 (13) | 15 (58)  11 (42)  - | 16 (55)  13 (45)  - | 41 (58)  28 (39)  2 (3) |
| **Blood group**  Match  Major mismatch  Minor mismatch  Bidirectional mismatch  Missing | 9 (56)  1 (6)  4 (25)  -  2 (13) | 13 (50)  7 (27)  4 (15)  1 (4)  1 (4) | 10 (34)  10 (34)  9 (31)  -  - | 32 (45)  18 (25)  17 (24)  1 (2)  3 (4) |

| **Graft versus host disease** |  |  |  |  |
| --- | --- | --- | --- | --- |
| **Development of GvHD** | 12(75) | 19 (73) | 25 (86) | 56 (79) |
| **Type of GvHD (n=56)**  Acute  Grade I & II  Grade III & IV  Unknown  Chronic  Unknown | 11 (92)  7 (64)  3 (27)  1 (9)  1 (8)  - | 15 (79)  13 (87)  2 (13)  -  4 (21)  - | 19 (76)  15 (79)  4 (21)  -  3 (12)  3 (12) | 45 (80)  35 (78)  9 (20)  1 (2)  8 (14)  3 (5) |
| **Location of acute GvHD (n=45)**  Skin only  Skin and other  Skin and intestine  Skin, intestine and other | 7 (64)  2 (18)  1 (9)  1 (9) | 10 (67)  2 (13)  3 (20)  - | 15 (79)  4 (21)  -  - | 32 (71)  8 (18)  4 (9)  1 (2) |
| **Location of chronic GvHD (n=8)**  Skin only  Skin and other | -  1 | 4  - | 1  2 | 5 (63)  3 (37) |

GvHD, graft versus host disease; HLA, human leukocyte antigen; ICCC-3, International Classification of Childhood Cancer, 3^rd^ edition; IQR, interquartile range; TBI, total body irradiation

^1^ Other tumors: tumor of the central nervous system (n=1)

^2^ Combination according to Cyclophosphamide Equivalent Dose (CED) (Green et al; Pediatr Blood Cancer. 2014 January; 61(1): 53–67. doi:10.1002/pbc.24679)

^3^ Thoracic radiation fields according to COG guidelines, Version 4.0, Oct 2018, including radiation to the chest, whole lung, mediastinum, (mini-)mantle field, TBI and additionally upper abdomen and thoracic spine, including craniospinal irradiation

^4^ Thoracic surgery according to COG guidelines, Version 4.0, Oct 2018, including thoracotomy, chest wall surgery, rib resection, lobectomy, pulmonary metastasectomy and wedge resection

**SUPPLEMENTAL TABLE S3:**

This supplemental table describes clinical, treatment and transplant characteristics of childhood cancer survivors transplanted autologous (N=61) stratified by era of transplantation

|  | **1976-1995**  (n=17) | **1996-2005**  (n=25) | **2006-2015**  (n=19) | **Total**  (n=61) |
| --- | --- | --- | --- | --- |
|  | n (%) | n (%) | n (%) | n (%) |
| **Clinical characteristics** |  |  |  |  |
| **Cancer diagnosis according to ICCC-3**  I: Leukemia  II: Lymphoma  IV: Neuroblastoma  Other^1^ | 2 (12)  8 (47)  5 (29)  2 (12) | 3 (12)  4 (16)  8 (32)  10 (42) | 0  5 (26)  6 (32)  8 (42) | 5 (8)  17 (28)  19 (31)  20 (33) |
|  |  |  |  |  |
| **Treatment characteristics** |  |  |  |  |
| **Conditioning containing TBI** | 5 (29) | 3 (12) | 1 (5) | 9 (15) |
| **Conditioning regimens** |  |  |  |  |
| TBI + cyclophosphamide ± others | 3 (18) | - | - | 3 (5) |
| TBI + others | 2 (12) | 3 (12) | 1 (5) | 6 (9) |
| Busulfan + cyclophosphamide ± other | 6 (35) | 2 (8) | 1 (5) | 9 (15) |
| Busulfan ± others | 1 (6) | 4 (16) | 2 (10) | 7 (11) |
| Lomustine ± others | 3 (18) | 3 (12) | 3 (16) | 9 (15) |
| Cyclophosphamide ± others | 1 (6) | 6 (24) | 2 (11) | 9 (15) |
| Melphalan ± Carboplatin ± others | 1 (6) | 7 (28) | 10 (53) | 18 (30) |
| **Chemotherapeutic agents** |  |  |  |  |
| Alkylating agents combined ^2^ | 17 (100) | 25 (100) | 19 (100) |  |
| Busulfan | 7 (41) | 7 (28) | 3 (16) | 17 (28) |
| Carmustine | 4 (24) | 3 (12) | 2 (11) | 9 (15) |
| Cyclophosphamide | 17 (100) | 21 (84) | 16 (84) | 54 (89) |
| Ifosfamide | 5 (29) | 15 (60) | 16 (84) | 36 (59) |
| Lomustine | 1 (6) | - | 1 (5) | 2 (3) |
| Melphalan | 9 (53) | 13 (52) | 15 (79) | 37 (61) |
| Thiotepa | 2 (12) | 7 (28) | 2 (11) | 11 (18) |
| Bleomycin | 2 (12) | 3 (12) | 2 (11) | 7 (11) |
| **Chemotherapeutic agents, mg/m^2^** |  |  |  |  |
| Alkylating agents combined ^2^ | 17164 (15921 – 18380) | 17286 (12650 – 35807) | 16522 (11894 – 31601) | 17154 (12650 – 31601) |
| Busulfan | 480 (470 – 600) | 480 (480 – 480) | 443 (374 – 470) | 480 (456 – 481) |
| Carmustine | 300 (298 – 351) | 300 (298 – 300) | 300 (291 – 306) | 300 (298 – 300) |
| Cyclophosphamide | 7454 (6970 – 9352) | 7491 (4000 – 9600) | 3385 (1704 – 4938) | 5980 (3357 – 8845) |
| Ifosfamide | 14000 (11500 – 16032) | 22500 (10000 – 44782) | 18903 (11628 – 55978) | 18038 (10113 – 49792) |
| Lomustine | 190 | - | 600 | 395 (190 – 600) |
| Melphalan | 140 (140 – 142) | 140 (140 – 179) | 140 (139 – 180) | 140 (140 – 179) |
| Thiotepa | 825 (750 – 900) | 894 (594 – 900) | 755 (610 – 900) | 894 (604 – 900) |
| Bleomycin | 41 (40 - 42) | 40 (40 - 50) | 30 (20 – 40) | 40 (40 – 42) |
| **Radiotherapy involving the thorax ^3^** | 10 (59) | 14 (56) | 11 (58) | 35 (57) |
| **Thoracic surgery ^4^** | 1 (6) | 4 (16) | 6 (32) | 11 (18) |
| **Transplant characteristics** |  |  |  |  |
| **Remission status at transplantation**  First remission  Relapsed disease | 8 (47)  9 (53) | 18 (72)  7 (28) | 11 (58)  8 (42) | 37 (61)  24 (39) |
| **Stem cell donor** |  |  |  |  |
| Autologous marrow | 8 (47) | 3 (12) | 0 | 11 (18) |
| Autologous apheresis | 9 (53) | 22 (88) | 19 (100) | 50 (82) |
| **Source of transplant** |  |  |  |  |
| Peripheral blood | 8 (47) | 22 (88) | 19 (100) | 49 (80) |
| Bone marrow | 9 (53) | 3 (12) | - | 12 (20 ) |

ICCC-3, International Classification of Childhood Cancer, 3^rd^ edition; IQR, interquartile range; TBI, total body irradiation

^1^ Other tumors: tumor of the central nervous system (n=5), retinoblastoma (n=1), malignant bone tumor (n=7), soft tissue sarcoma (n=4), germ cell tumors (n=3)

^2^ Combination according to Cyclophosphamide Equivalent Dose (CED) (Green et al; Pediatr Blood Cancer. 2014 January; 61(1): 53–67. doi:10.1002/pbc.24679)

^3^ Thoracic radiation fields according to COG guidelines, Version 4.0, Oct 2018, including radiation to the chest, whole lung, mediastinum, (mini-)mantle field, TBI and additionally upper abdomen and thoracic spine, including craniospinal irradiation

^4^ Thoracic surgery according to COG guidelines, Version 4.0, Oct 2018, including thoracotomy, chest wall surgery, rib resection, lobectomy, pulmonary metastasectomy and wedge resection

**SUPPLEMENTAL TABLE S4**:

This supplemental table compares clinical, treatment and transplant characteristics of childhood cancer survivors after allogeneic and autologous hematopoietic stem cell transplantation, N=132, 52%male

|  | **Allogeneic HSCT**  **N=71** | **Autologous HSCT**  **N=61** | **p value^1,2^** |
| --- | --- | --- | --- |
|  |  |  |  |
|  | n (%) | n (%) |  |
| **Sociodemographic characteristics** |  |  |  |
| **Sex**, male | 38 (54) | 31 (51) | 0.757 |
| **Age at survey,** median years (IQR) | 18.5 (13.8 – 23.5) | 18.3 (13.8 – 22.6) | 0.688 |
| **Smoking status**  Active smoking  Passive smoking  Former active smoking  Never active smoking | 4 (6)  30 (42)  6 (8)  31 (44) | 3 (5)  26 (43)  3 (5)  29 (47) | 0.863 |
| **Clinical characteristics** |  |  |  |
| **Age at diagnosis,** median years (IQR) | 7.1 (3.2 - 11.1) | 5.6 (2.6 – 12.9) | 0.731 |
| **Age at diagnosis,** years  0-4  5-9  10-14  15-21 | 26 (37)  27 (38)  15 (21)  3 (4) | 27 (44)  12 (20)  15 (25)  7 (11) | 0.083 |
| **Age at first HSCT,** median years (IQR) | 8.7 (5.7 - 13.0) | 9.6 (3.1 – 14.3) | 0.953 |
| **Follow-up time,** median years (IQR) | 10.2 (7.4 - 16.1) | 9.5 (7.2 – 15.8) | 0.420 |
| **Time to HSCT,** median years (IQR) | 0.9 (0.4 – 2.8) | 0.7 (0.5 – 1.9) | 0.511 |
| **Era of HSCT**  1976 - 1995  1996 - 2005  2006 - 2015 | 16 (22)  26 (37)  29 (41) | 17 (28)  25 (41)  19 (31) | 0.501 |
| **Cancer diagnosis according to ICCC-3**  I: Leukemia  II: Lymphoma  IV: Neuroblastoma  Other^3^ | 67 (94)  3 (4)  0  1 (2) | 5 (8)  17 (28)  19 (31)  20 (33) | <0.005 |
|  |  |  |  |
| **Treatment characteristics** |  |  |  |
| **Conditioning regimens**  TBI + cyclophosphamide ± others  TBI + others  Busulfan + cyclophosphamide ± others  Busulfan ± others  BCNU ± others  CYC ± others  Melphalan ± Carboplatin ± others | 31 (44)  19 (27)  19 (27)  1 (1)  -  -  1 (1) | 3 (5)  6 (10)  9 (15)  7 (11)  9 (15)  9 (15)  18 (29) | <0.001 |
| **Chemotherapeutic agents**  Alkylating agents combined ^7^  Busulfan  Carmustin  Cyclophosphamide  Ifosfamide  Lomustin  Melphalan  Thiotepa  Bleomycin | 70  21  0  69  26  0  7  3  1 (1) | 61  16  10  54  36  2  37  11  7 (12) |  |
| **Chemotherapeutic agents, mg/m^2^ ^3^**  Alkylating agents combined ^7^  Busulfan  Carmustin  Cyclophosphamide  Ifosfamide  Lomustin  Melphalan  Thiotepa  Bleomycin | 6,100 (3871 - 9579)  360 (320 - 445)  -  3990 (2983 - 6242)  4011 (3879 - 6126)  -  140 (139-140)  304 (168 - 307)  80 | 17,154 (12,650 – 31,601)  480 (456 - 481)  300 (298-300)  5980 (3357 - 8845)  18,038 (10,113-49,792)  395 (190-600)  140 (140-179)  894 (604 - 900)  40 (40-42) | <0.001  0.0038  na  0.01  0.0001  na  0.193  <0.001  na |
| **Radiotherapy involving the thorax ^8^**  Yes  No | 51 (72)  20 (28) | 34 (54)  27 (44) | 0.054 |
| **Thoracic surgery ^9^**  Yes  No | 1 (1)  70 (99) | 11 (18)  50 (82) | 0.364 |
|  |  |  |  |
| **Transplant characteristics** |  |  |  |
| **Remission status at transplantation**  First remission  Relapsed disease | 38 (54)  33 (46) | 37 (61)  24 (39) | 0.409 |

HSCT, hematopoietic stem cell transplantation; IQR, interquartile range

^1^ p-value calculate by Pearson chi-square to compare categorical variables between allogeneic and autologous transplant cohort

^2^ p-value calculate by t-test to compare categorical variables between allogeneic and autologous transplant cohort

^4^ body mass index (BMI) defined as normal if z-score ≥-2 and ≤1 (responder ≤19) or kg/m^2^ ≥18.5 and ≤24.9 (responder >19 years)

^6^ Relation sex of donor to recipient: match= both male or female; mismatch= donor male and recipient female or vice versa

^7^ Combination according to Cyclophosphamide Equivalent Dose (CED) (Green et al; Pediatr Blood Cancer. 2014 January; 61(1): 53–67. doi:10.1002/pbc.24679)

^8^ Thoracic radiation fields defined according to COG guidelines, Version 4.0, Oct 2018

^9^ Relevant thoracic surgery defined according to COG guidelines, Version 4.0, Oct 2018

**SUPPLEMENTAL TABLE S5**:

This supplemental table describes the characteristics of childhood cancer survivors who have been treated with hematopoietic stem cell transplantation and thoracic surgery, N=12

|  | **Diagnosis** | **Year of diagnosis** | **Age at first thoracic surgery [years]** | **Relapse** | **Description of thoracic surgery/ surgeries** | **Lung toxic exposure** |
| --- | --- | --- | --- | --- | --- | --- |
| 1 | Hodgkin lymphomas | 1986 | 10 | Yes | Thoracotomy: tumor resection mediastinal | BCNU, Bleomycin, CCNU, CYC, Mel  Radiotherapy: mediastinal, lung |
| 2 | Ewing sarcoma | 1997 | 26 | Yes | Thoracoscopy: metastasectomy | CYC, IFO, Mel |
| 3 | Non-Hodgkin lymphomas | 1996 | 17 | Yes | Thoracotomy: lobectomy upper lobe and lingual left side | Busulfan, CYC  Radiotherapy: mediastinal |
| 4 | Acute myeloid leukemias | 1999 | 5 | Yes | Thoracoscopy: lobectomy left lower lobe due to infection | Radiotherapy: TBI, mediastinal |
| 5 | Ewing sarcoma | 2002 | 14 | No | 1x Thoracoscopy: metastasectomy  3x Thoracotomy: 2x Metastasectomy and resection left lower lobe | Busulfan, CYC, IFO, Mel  Radiotherapy: lung |
| 6 | Ewing sarcoma | 2004 | 13 | Yes | Thoracotomy: lobectomy left lower lobe and parietal pleura  Thoracotomy: extended extrapleural residual pneumonectomy  Thoracic wall: partial scapula resection | Busulfan, IFO, Mel  Radiotherapy: lung |
| 7 | Ewing sarcoma | 2004 | 5 | Yes | Thoracotomy: subtotal tumor resection  Thoracotomy: marginal tumor resection, resection of two ribs  Thoracotomy: metastasectomy, partial resection right upper lobe | CYC, IFO, Mel  Radiotherapy: lung, chest wall |
| 8 | Ewing sarcoma | 2006 | 4 | Yes | Thoracoscopy (VATS): two lung biopsies | Busulfan, CYC, IFO, Mel |
| 9 | Non-Hodgkin lymphomas | 2006 | 13 | No | Sternotomy and mediastinotomy: tumor biopsy  Sternotomy: partial tumor resection | Busulfan, CYC, IFO  Radiotherapy: mediastinal |
| 10 | Hodgkin lymphomas | 2007 | 17 | Yes | Thoracotomy: tumor resection | BCNU, Bleo, CYC, IFO, Mel  Radiotherapy: mediastinal |
| 11 | Ewing sarcoma | 2008 | 10 | Yes | Thoracotomy and resection of two ribs | CYC, IFO, Mel  Radiotherapy: thoracic spine |
| 12 | Ewing sarcoma | 2008 | 19 | Yes | Bilateral VATS: metastasectomy | CYC, IFO, Mel  Radiotherapy: lung |

BCNU, carmustin; CCNU, lomustin; CYC, cyclophosphamide; IFO, ifosfamide; Mel, melphalan;


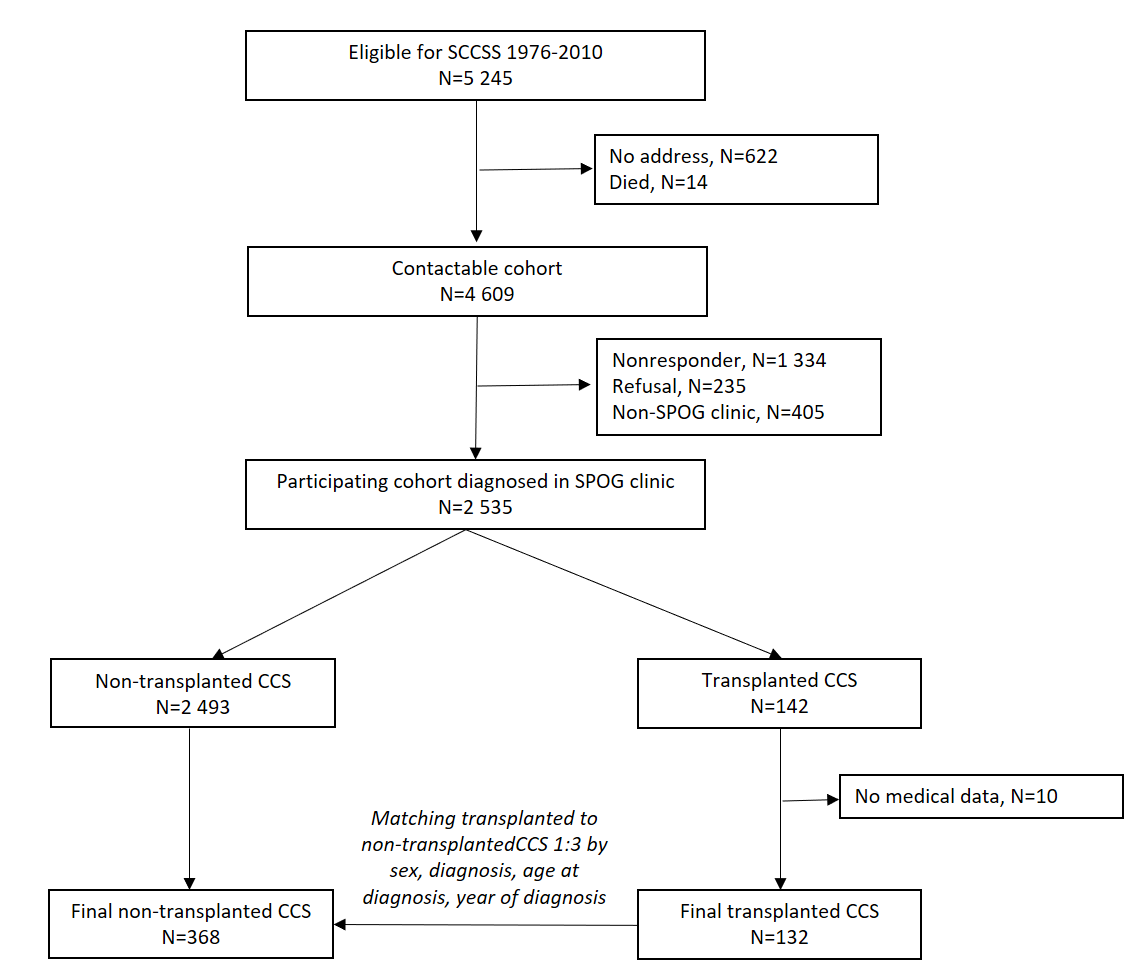


## **SUPPLEMENTAL FIGURE F1**: Population tree of transplanted and non-transplanted childhood cancer survivors eligible for this study – approach 1 with division into transplanted and non-transplanted survivors at the end.

CCS, childhood cancer survivor; SPOG, Swiss Pediatric Oncology Group


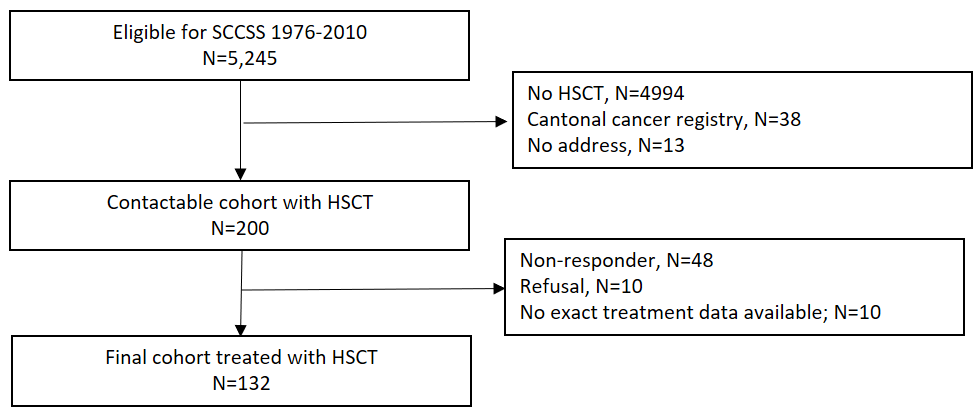


## **SUPPLEMENTAL FIGURE F2**: Population tree of transplanted childhood cancer survivors eligible for this study – approach 2 with division into transplanted and non-transplanted survivors at the beginning.

CCS, childhood cancer survivor; HSCT, Hematopoietic Stem Cell Transplantation
